# Zika infection decreases *Aedes aegypti* locomotor activity but does not influence egg production or viability

**DOI:** 10.1590/0074-02760180290

**Published:** 2018-08-23

**Authors:** Karine Pedreira Padilha, Maria Eduarda Barreto Resck, Octávio Augusto Talyuli da Cunha, Rayane Teles-de-Freitas, Stéphanie Silva Campos, Marcos Henrique Ferreira Sorgine, Ricardo Lourenço-de-Oliveira, Luana Cristina Farnesi, Rafaela Vieira Bruno

**Affiliations:** 1 Fundação Oswaldo Cruz-Fiocruz, Instituto Oswaldo Cruz, Laboratório de Biologia Molecular de Insetos, Rio de Janeiro, RJ, Brasil; 2 Universidade Federal do Rio de Janeiro, Instituto de Bioquímica Médica Leopoldo de Meis, Laboratório de Bioquímica de Insetos Hematófagos, Rio de Janeiro, RJ, Brasil; 3 Fundação Oswaldo Cruz-Fiocruz, Instituto Oswaldo Cruz, Laboratório de Mosquitos Transmissores de Hematozoários, Rio de Janeiro, RJ, Brasil; 4 Conselho Nacional de Desenvolvimento Científico e Tecnológico, Instituto Nacional de Ciência e Tecnologia em Entomologia Médica, Brasil

**Keywords:** Zika virus, Aedes aegypti, locomotor activity, egg production, egg viability

## Abstract

**BACKGROUND:**

Zika has emerged as a new public health threat after the explosive epidemic in Brazil in 2015. It is an arbovirus transmitted mainly by *Aedes aegypti* mosquitoes. The knowledge of physiological, behavioural and biological features in virus-infected vectors may help the understanding of arbovirus transmission dynamics and elucidate their influence in vector capacity.

**OBJECTIVES:**

We aimed to investigate the effects of Zika virus (ZIKV) infection in the behaviour of *Ae. aegypti* females by analysing the locomotor activity, egg production and viability.

**METHODOLOGY:**

*Ae. aegypti* females were orally infected with ZIKV through an artificial feeder to access egg production, egg viability and locomotor activity. For egg production and viability assays, females were kept in cages containing an artificial site for oviposition and eggs were counted. Locomotor activity assays were performed in activity monitors and an average of 5th, 6th and 7th days after infective feeding was calculated.

**FINDINGS:**

No significant difference in the number of eggs laid per females neither in their viability were found between ZIKV infected and non-infected females, regardless the tested pair of mosquito population and virus strain and the gonotrophic cycles. Locomotor activity assays were performed regardless of the locomotor activity in ZIKV infected females was observed, in both LD and DD conditions.

**MAIN CONCLUSIONS:**

The lower locomotor activity may reduce the mobility of the mosquitoes and may explain case clustering within households reported during Zika outbreaks such as in Rio de Janeiro 2015. Nevertheless, the mosquitoes infected with ZIKV are still able to disseminate and to transmit the disease, especially in places where there are many oviposition sites.

The Zika virus (ZIKV) is an arbovirus belonging to the *Flaviviridae* family, first isolated in 1947 in Uganda, West Africa from a sentinel rhesus monkey.^1^ Since then, sporadic human cases were reported in Asia and Africa. The first large Zika epizooty reported occurred in Micronesia in 2007.^2^

ZIKV was previously believed to cause only a mild and self-limiting illness, but it has emerged as a new public health threat after the explosive epidemic in Brazil in 2015, specially due to the increased severe congenital malformations (microcephaly) and neurological complications reported in the country.^3^ This new epidemiological scenario led the World Health Organization to declare the Zika epidemic as a Sanitary Emergency in Public Health in February of 2016.

ZIKV is transmitted by several species of mosquitoes of the family Culicidae, mainly *Aedes aegypti*.^4^ Besides being the single confirmed natural ZIKV vector during the Pan-American epizootic,^5^ *Ae. aegypti* is also the only vector for several other arboviruses circulating in the New World, such as the four Dengue virus (DENV) serotypes,^6^ Chikungunya virus^7^ and Yellow Fever virus^8^.

The knowledge of the physiological, behavioural and biological features of the vector, such as preference and frequency of haematophagy in humans, egg laying, gonotrophic discordance and resistance to desiccation^9^ may help the understanding of arbovirus transmission dynamics and elucidate the role of those parameters impacting vector capacity.

The haematophagous and anthropophagic behaviours are crucial to females’ egg maturation. A single *Ae. aegypti* female can lay from 100 to 200 eggs per batch, and multiple times throughout her lifetime after each blood meal.^10^ Eggs are laid usually on container surfaces near water, preferentially in shaded places^9^ and their embryonic development and egg viability is directly related to environmental temperature^11^.

Concerning activity patterns mosquito species are classified as diurnal, crepuscular and nocturnal.^12^ *Ae. aegypti* is considered a diurnal and crepuscular species that can modify the activity pattern according to changes in the physiology and viral infection.^12^ Accordingly, DENV-2 artificially-infected *Ae. aegypti* females showed increased locomotor activity, potentially effecting the infection kinetics and disease transmission.^13^ Moreover, the number of eggs laid by *Ae. aegypti* females orally challenged with DENV-2 may vary during the mosquito lifetime, with a decrease from one gonotrophic cycle to another.^14^

Here, we aimed to investigate the effects of ZIKV infection in the behaviour of *Ae. aegypti* females by analysing the locomotor activity, egg production and egg viability.

## MATERIALS AND METHODS

*Mosquito populations and rearing* - Eggs from *Ae. aegypti* mosquitoes (strain PAEA, Tahiti, French Polynesia) were hatched in plastic trays containing 1.5L of Milli-RO water and approximately 1g of yeast (two tablets of Vitalab®, Brazil). Larvae were fed with the same quantity of yeast, every two days, until pupae development according to Farnesi et al.^11^ The pupae were counted and separated in cages (with approximately 400 each) for adult emergence. Males and females mosquitoes were kept together in cages with 10% sucrose solution *ad libitum*. In all experiments, rearing of mosquitoes was carried out in an incubator (Precision Scientific Incubator, USA) at 25ºC, in a photoperiod of 12 hours of light and dark (LD 12:12) and 60-80% relative humidity.

*Virus and experimental infection* - The ZIKV strain ZIKV/H.sapiens/Brazil/PE243/201 (GenBank accession number KX197192.1) was used for oral experimental infection of *Ae. aegypti* females as previously described.^15^ The virus was previously isolated from a febrile patient in the state of Pernambuco and molecularly characterised.^16^ Briefly, females were allowed to feed through a membrane attached to an artificial feeder kept at 37ºC for approximately 40 min inside a Biosafety level - 2 (BSL-2) insectary facility. The infectious blood meal consisted of 1:1 mix of rabbit red blood cells and L-15 culture medium containing ZIKV at a final concentration of 10^7^ PFU/mL; ATP pH 7.4 at a final concentration of 1 mM was included as a phagostimulant. Control mosquitoes also fed with a similar blood meal, but with a non-infected L15 culture medium.^15^ After blood meal, mosquitoes were cold-anesthetised and only the fully engorged females were considered. Viral detection was done by polymerase chain reaction (PCR) (see Supplementary data, Fig. 1, Supporting Information).

*Egg production assays in ZIKV infected and uninfected mosquitoes* - For gonotrophic cycle assays, approximately 450 females, around two weeks-old, were used per condition (infected or uninfected). We performed three replicates per experiment; each one contained, at least, 50 females that were deprived of sugar prior to one infected or uninfected blood meal (with *Swis*s mice or rabbit blood), for approximately 40 min. In both cases, infected or uninfected blood meal, engorged females mosquitoes were selected (at least, 150 females, in each condition, were used per experiment). After three days, the oviposition was stimulated in cages containing an artificial site for oviposition: plastic container containing 100 mL of filtered water and three strips of rectangular filter paper, 8 cm x 15 cm in size, for two days. In these assays we used the second and third gonotrophic cycles. Three independent experiments were performed.

*Eggs viability assays* - For eggs viability analysis, the eggs obtained as described above were removed from the oviposition site and placed to dry in a humid chamber. After one week drying, eggs were removed carefully from the filter paper using a brush and counted in the Egg Counter Program (©BioAlg Group, Faro, Portugal). Afterwards, eggs were randomly selected and tested for viability as described below.

Each replica was set up with 50 counted eggs and placed on filter paper to stimulate hatching in Petri dishes containing 50 mL of industrial yeast extract solution 0.15% (weight/volume) for 24 h in a Precision Scientific Incubator (Thermo Fischer) under a constant temperature of 25ºC and 60-80% relative humidity according to Farnesi et al.^11^ We analysed eggs viability from the second and third gonotrophic cycles. Each viability experiment analysed contained 600 eggs (300 per gonotrophic cycle, being 150 from uninfected and 150 from ZIKV infected females). This assay was composed by three independent experiments, totalising 1,800 eggs.

*Egg production assays in ZIKV infected and uninfected mosquitoes in the other pair vector/virus* - In addition, we verified if egg production or viability records would be similar in other pair vector/virus: *Ae. aegypti* (Urca population) and ZIKV (Rio-S1 strain, GenBank accession number KU926310), both originated from Rio de Janeiro and whose vector competence parameters have been previously described to be considerably high.^17^ All mosquito treatments, virus titter in the blood meal and other experimental infection procedures were as described above, except that mosquito females from Urca population took a second uninfected blood meal 14 days after being orally challenged by ZIKV. Assessments of egg production or viability was limited to the second gonotrophic cycle.

*Locomotor/flight activity assays* - *Ae. aegypti* females, around 15 days post emergence, were transferred to four small circular carton cages (60 per cage) lined with micro tulle (8.5 cm of diameter X 9.5 cm of height). Females were deprived of sucrose solution for approximately 10 h prior to a blood meal (ZIKV infected or uninfected blood). Blood feeding followed as described above. ZIKV infected (n *=* 51) and non-infected *Ae. aegypti* females (n *=* 54) were individualised in 25 mm glass tubes to analyse locomotion and flight activity. In these tubes, cottons soaked with 10% sucrose solution were placed in one end and the other end was sealed with parafilm. Mosquito tubes were put in locomotor activity monitors (Trikinetics Inc, Waltham, MA, USA) with 32 channels and infrared light beams, where mosquitoes movement could be detected and recorded each time the mosquito passed through the infrared beam.

The experiment lasted, at least, eight days to allow viral dissemination according to Ryckebusch et al.^18^ The monitors were kept inside an incubator (Precision Scientific Incubator, USA) at constant temperature of 25^o^C, in a LD 12:12 regimen (12 h of light followed by 12 h of dark), during seven days and a DD regimen (24 h of constant dark), for one day. The relative humidity was 60-80%. To analyse the locomotor activity, we did an average of 5rd, 6th and 7th days of activity, corresponding to LD condition and 8th DPI, corresponding to a day in DD condition. We performed three independent experiments, with a total of 123 ZIKV infected and 122 control mosquitoes analysed but here we show a representative data with 54 ZIKV infected and 51 control females.

The results were organised and analysed in Excel (Microsoft Office) with parameters previously established^19^ and only mosquitoes that were alive at the end of the experiment and positive for ZIKV were considered (See Supplementary data, Fig. 1).

*Statistical analysis* - The locomotor activity results were analysed, firstly for Shapiro-Wilk normality test. After, we used the parametric *t-*Student test considering the log (N+1) mean of the individual mosquito data every 30 min. Since the mosquito activity data is especially variable, the transformation of the data to logarithm allows their distribution to be more constrained and the average to be less influenced by very low or very high values. In fact, because we have many zeros in the data series, we must use log (N+1) instead of log N. The advantage of using this calculation is that it prevents the masking of data by the effect of very high numbers within a single interval.^20^ In the analysis of egg quantification and viability, we first performed the Shapiro-Wilk normality test. When the data showed a normal distribution, we used the parametric *t-*student; when data showed a non-normal distribution, we used non-parametric Mann-Whitney test. Other specific statistical information’s are in the figure legend. All analysis were performed using GraphPad Prism 5 (GraphPad Software, San Diego, California, USA) and p value < 0,05 considered statistically significant.

*Ethical statement* - All the experiments carried out on this study were approved by the institutional Research Ethics Committees IOC/FIOCRUZ #LW34/14 (for feeding on mice) and CEUA-UFRJ 155/13 (for use of rabbit blood).

## RESULTS

**Fig. 1:**
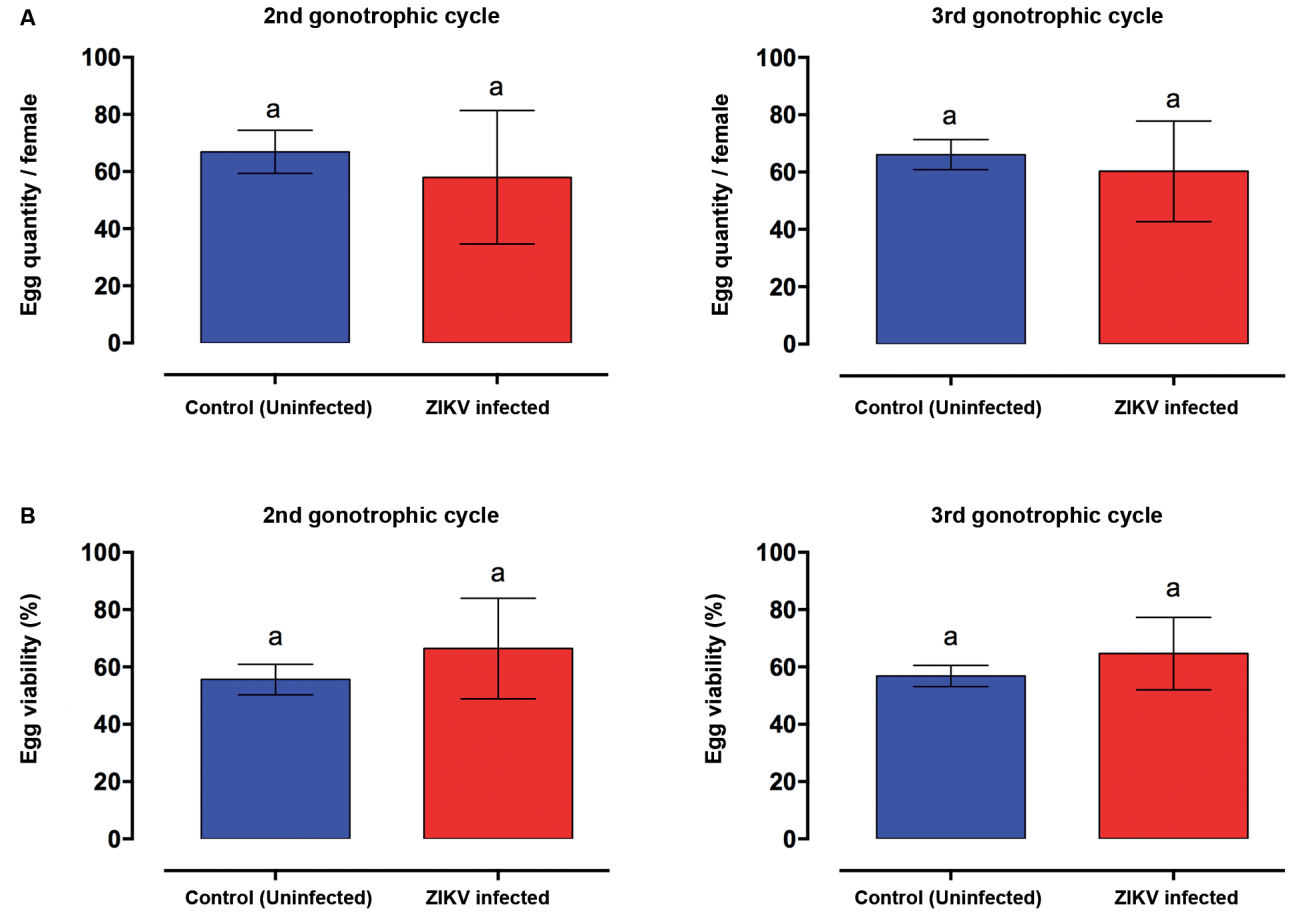
effect of Zika virus (ZIKV) infection on the eggs quantity (A) and viability (B) of PAEA *Aedes aegypti* females infected with ZIKV PE243, according to the second and third gonotrophic cycles. The lack of significance is represented by p values > 0.05 obtained by using the parametric *t*-Student and non-parametric Mann-Whitney tests, respectively. Error bars represent mean ± s.d of three independent experiments.

*ZIKV infection has no effect on egg quantity and viability* - All ZIKV orally challenged mosquitoes used in this study tested positive (Supplementary data, Fig. 1). To determine whether ZIKV infection affects *Ae. aegypti’s* egg production and viability, the second and third gonotrophic cycles were analysed. The overall results showed that, regardless of the gonotrophic cycle (Supplementary data, Fig. 2) and the pair mosquito-virus strain (Supplementary data, Fig. 3), no difference was observed in egg production and viability for ZIKV infected and non-infected *Ae. aegypti* females. Similarly, when we analysed the second and third gonotrophic cycles comparatively, no significant differences were observed either in quantity (p = 0.4091 and p = 0.3496, respectively) or viability (p = 0.0773 and p = 0.0734, respectively) (Fig. 1).

**Fig. 2:**
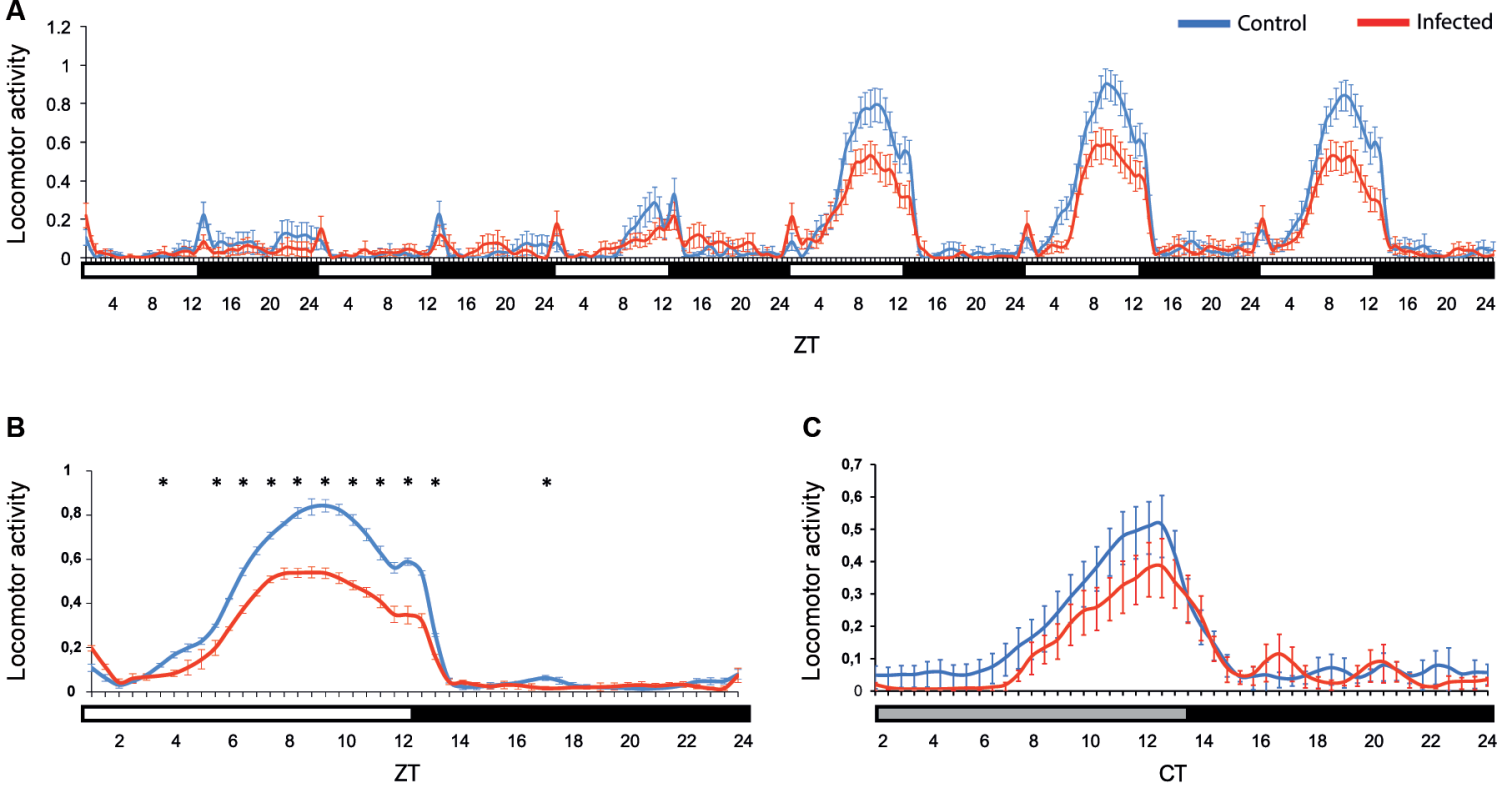
locomotor activity of *Aedes aegypti* females infected by Zika virus (ZIKV). Locomotor activity of control (blue line, n *=* 51) and infected *Ae. aegypti* females (red line, n *=* 54). The mosquitoes were observed from the second to the seventh days post infection (DPI) in LD12:12 (A). The first DPI was not included because we considered them to be still adapting to the system. We also show an average profile in LD (the graph referred only to 5th, 6th and 7th DPI) (B). On the 8th DPI the mosquitoes were kept in constant darkness (DD) (C). Bars below the graphics indicate the light regime: white = lights on in LD cycles, grey = lights off in DD (“subjective day”), black = lights off in LD or DD (“subjective night”). ZT: *Zeitgeber* time within a light/dark cycle experiment; ZT0: time the light turns on; ZT12: time the light turns off; CT: circadian time in DD. Error bars were shown for each 30 min interval. Asterisks represent the significance of the *t*-Student test, where p < 0.05.

*ZIKV infection decreases females Ae. aegypti locomotor activity* - To evaluate if ZIKV infection could disrupt the diurnal activity pattern of the females, we tested infected and non-infected females for seven days under a LD12:12 cycle. In the first two days of the assay, the activity was very low, probably because of blood digestion. The females became more active from the fourth day on. We focused our analysis on the 5th, 6th and 7th days post infection for a better interpretation. They present a low intensity startle response not controlled by the endogenous clock when lights turn on, which is commonly seen in locomotor activity tests (ZT 0.5, Fig. 2A-B). Comparatively, during the two peaks activity at ZT9 and ZT12, infected *Ae. aegypti* females showed significantly lower locomotor activity than the uninfected ones (a clear difference persisted at ZT5 - ZT13; p < 0.05) (Fig. 2B). In both infected and control groups, the major activity peak occurs between ZT 6 to 10 in LD condition. On the 8th day post infection, the mosquitoes went to a constant dark regimen (DD), to verify the influence of the endogenous circadian clock on this altered activity. Contrary to what was observed in LD, in DD conditions the startle response was eliminated, as expected (Fig. 2C). We only observed one activity peak at CT11 (Fig. 2C). The infected females in DD also presented less activity compared to the control ones, although the difference was not statistically significant in this case. After the peak, the activity was drastically reduced in both groups in LD and DD conditions (Fig. 2).

## DISCUSSION

Since the Zika outbreak and all the consequences caused by the virus infection in newborns, mainly in Brazil, the major efforts of the scientific community have been focused on the interaction between virus and the vertebrate vector (humans). However, we believe it is also fundamental to investigate in detail the effects of this virus in the insect vector. We aimed to contribute to a major understanding of possible changes caused in the *Ae. aegypti* females behaviour when infected with ZIKV, which will inevitably influence the success of vector control measures.

Recently, *Ae. aegypti* was reported naturally infected by ZIKV.^5^ Field and laboratory studies to assess the transmitting success of such an arbovirus to a new host and their offspring are still scarce. Here, we analysed fecundity (i.e., the number of eggs laid by each female) and fertility (the number of viable offspring produced) as well as locomotor activity in ZIKV infected *Ae. aegypti*.

In fecundity analysis, there was no difference between ZIKV infected and uninfected mosquitoes, regardless of the tested pair of mosquito population and virus strain. However, a borderline p value in the statistical analysis (p = 0.054) suggests a trend for higher average viability in eggs from infected females (Fig. 1, Supplementary data, Figs 2-3). In fecundity analysis, there were no differences between ZIKV infected and uninfected mosquitoes, even when using another laboratorial approach.^21^ In contrast, a previous study showed that DENV infected *Ae. aegypti* females exhibited lower fecundity (egg quantity).^14^ Furthermore, lower egg production and hatching were observed when *Ae. aegypti* females were infected by DENV-1 or DENV-2.^14^ ^,^ ^22^ Therefore, our data suggest that the ZIKV infection does not cause damage to the fertility and viability in its main vector *Ae. aegypti*.

The locomotor activity has an important role in arboviruses spread and transmission dynamics. It was previously observed that DENV-2 infection causes an increase in locomotor activity in *Ae. aegypti* during the 24-h period in a LD 12:12 cycle.^13^ On the other hand, different alterations in mosquito physiology, like insemination and blood feeding, can also decrease the locomotor activity of *Ae. aegypti* and *Ae. albopictus*.^23^ However, different from our previous study with locomotor activity,^13^ we performed experimental mosquito infection via oral artificial feeding to mimic as closely as possible the natural kinetics of ZIKV infection and dissemination.

Recently, Ryckebusch et al.^18^ showed in the *Ae. aegypti* PAEA strain that the dissemination of the ZIKV occurs between 6th days post infection and 14th days post infection. In our analysis, we consider the interval between 5th and 8th days post infection to test the locomotor activity, to ensure both viral dissemination through the body and mosquito survival.

The very low activity soon after blood feeding was already previously described in mosquitoes and it is independent of the viral infection. It is related to blood metabolism, necessary to perform the oviposition, which normally occurs three days after the blood meal.^12^

The females of both groups started to exhibit a clear diurnal pattern of activity from the fourth day on. In addition to the traditional activity peak at ZT12 in LD, our mosquitoes also showed a peak at ZT9. Interestingly, this seems to be a characteristic of PAEA, since Lima-Camara et al.^13^ had already observed an increase in activity near ZT9 in this strain of *Ae. aegypti*. However, ZIKV infection in the *Ae. aegypti* PAEA strain caused a decrease in the activity pattern during the whole light phase in comparison to the control group throughout the analysed days in LD condition (Fig. 2A-B). This is the opposite of what Lima-Camara et al.^13^ observed for mosquitoes infected with DENV-2. Thus, it is possible that these viruses influence the behaviour of *Ae. aegypti* by different molecular targets. Furthermore, in DD condition, which means an absence of environmental conditions, both ZIKV infected and uninfected females presented one peak at CT11 (Fig. 2C). Since *Ae. aegypti* has an activity period of approximately 22-23h in DD,^19^ it is likely that the peak observed at ZT12 in LD has advanced to CT11 under constant conditions (DD). On the other hand, the observed peak at ZT9 in LD does not present a corresponding peak in DD, an indication that it could be controlled by a masking effect, free from the influence of the clock (for more details regarding the masking mechanisms see Clements^12^).

However, although our locomotor activity data suggest circadian clock involvement, it is possible that Zika infection does not initially need to recruit the central clock in the brain to produce the observed effects. That is because our experiments was continued up until to the ninth activity day (8th day post infection) and according to Ryckebusch et al.,^18^ virus dissemination to head tissues and salivary glands occurs only from the 14th day post infection. Insects have central and peripheral clocks. The former are responsible for directing the main circadian behaviours and the others are located in several tissues of the body, being fundamental for the modulation of the central clock.^12^ Microarray studies estimate that thousands of mosquito genes are controlled in the head and body by circadian clocks.^24^ ^,^ ^25^ Thus, it is quite feasible that Zika infection is able to modulate the behaviour of vectors by the influence of peripheral clocks in the initial days of infection.

Lately, Gaburro and collaborators^21^ using microelectrodes to record electrical activity in mosquito primary neurons culture and pools of females to analyse the locomotor activity in Zika infected mosquitoes, showed an increase in spiking activity of the neuronal network and in diurnal locomotion activity compared to uninfected females. Our results are quite different from those found recently by Gaburro et al.^21^ It is worthy to mention that we used a validated approach in behaviour studies^13^ ^,^ ^19^ ^,^ ^20^ ^,^ ^23^ in which the individual insects are isolated from host odours and inter-specimens communication. These cues may cause a bias and interfere in the overall activity of mosquitoes.

It is noteworthy that the ZIKV has a specific characteristic not described in other viruses of the same family: high tropism by brain tissues of the mosquito.^21^ Moreover, when the transcriptome of Zika infected mosquitoes is compared to DENV infected ones, the majority of mosquito genes (61%) that presented a modification in expression (up- or downregulation) are those of Zika infected group. These data suggest that there is a remarkable difference in the mosquito response to these viruses, which could lead to very different physiological and behavioural responses.^26^

The occurrence of Zika epidemics in recent years may be due to several factors, and the non-vector borne forms of transmission cannot be excluded, like in different humans fluids.^27^ In Brazil, another determinant may greatly influence the success of ZIKV transmission and maintenance, such as mosquito behaviour, climatic and environmental factors.^28^ Moreover, the high mosquito infestation index^29^, abundant vector breeding sites and deteriorated infrastructure may also be influencing the transmission of diseases, such as dengue and Zika.^30^ Our data show that Zika infection affects neither egg production nor viability and decreases mosquito locomotor activity. These alterations do not seem to negatively influence Zika transmission, once the majority of positive cases tested in the 2015 outbreak in Rio de Janeiro clustered within households.^30^ Thus, despite the lower activity, mosquitoes infected with ZIKV are still able to disseminate and transmit the disease, keeping the population, especially because their fertilily and fecundity are not alterated for this infection.
