# Supplementary material for: Zika infection decreases Aedes aegypti locomotor activity but does not influence egg production or viability
Source: Mem Inst Oswaldo Cruz. 2018 Aug 23;113(10):e180290. doi: 10.1590/0074-02760180290 (PMC6107100; doi:10.1590/0074-02760180290)
Supplement: Supplementary file 1 [file 1678-8060-mioc-113-10-e180290-s.pdf]

Supporting information

Molecular detection of ZIKV in mosquitoes - The viral RNA was extracted from the whole body using QIAmp Viral Mini Kit (Qiagen, Germany) according to manufacturer's protocol for molecular detection of ZIKV in the experimentally infected *Aedes aegypti* females. The RT-PCR for detecting ZIKV RNA was conducted using the Zika 4481- 5'CTGTGGCATGAACCCAATAG3' and Zika 4552c - 3' ATCCCATAGAG-CACCACTCC5' primers[1] in a Veriti 96 well Thermocycler model 9902 (Applied Biosystems, Foster City, California, USA), as previously described,[2] except for the pair Urca *Ae. aegypti* - Rio S-1 ZIKV strain where we used a RT-qPCR which details are available elsewhere.[3]

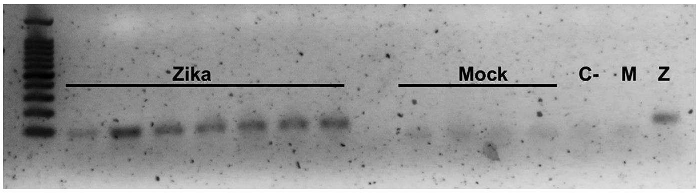

Fig. 1: Zika virus (ZIKV) detection by real time-polymerase chain reaction (RT-PCR). Whole body of infected mosquitoes were collected seven days after the feeding and a PCR reaction was performed to confirm the Zika infection. The first seven lanes showed the infected mosquitoes and the following four lanes showed the control (mock). The "C" represents the reaction without any cDNA template, while "M" and "Z" are mock and Zika C6/36 supernatant controls, respectively.

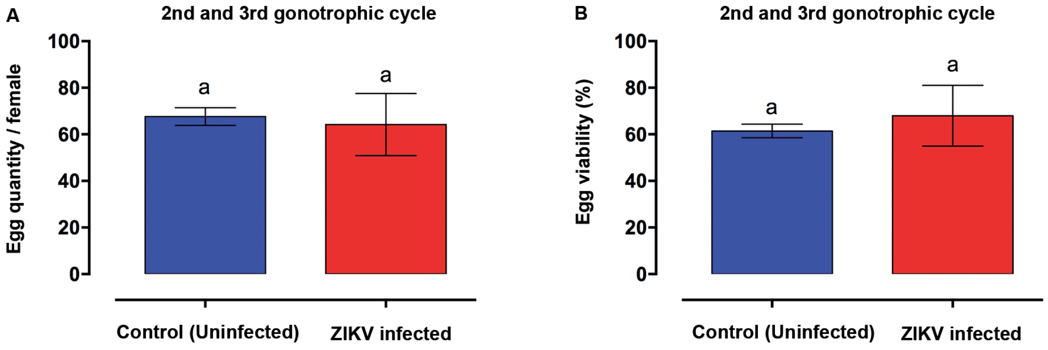

Fig. 2: overall effect of Zika virus (ZIKV) infection on the eggs quantity (A) and viability (B) of *Aedes aegypti* females. The lack of significance is represented by p values > 0.05 obtained by the parametric *t*-Student and non-parametric Mann-Whitney tests, respectively. Error bars represent mean ± s.d of three independent experiments. No significant differences were found neither in eggs production (p = 0.3699) nor viability (p = 0.5757).

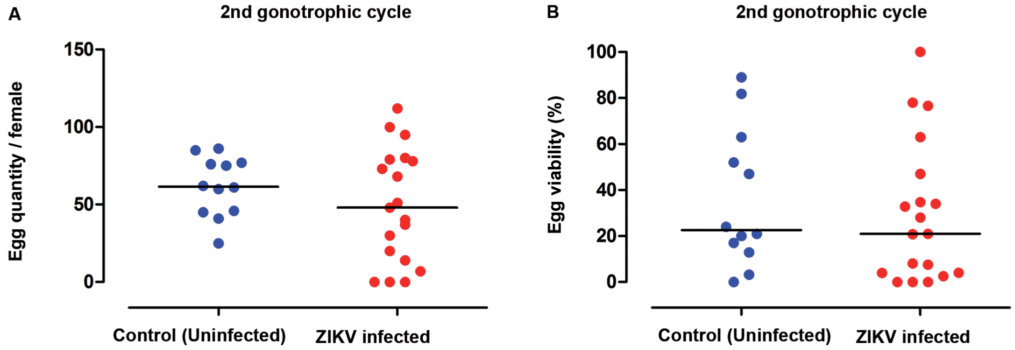

Fig 3: effect of Zika virus (ZIKV) infection on the eggs quantity (A) and viability (B) of *Aedes aegypti* females (Urca population) in the second gonotrophic cycle. Females took a second uninfected bloodmeal 14 days after being orally challenged by the Rio-S1 ZIKV strain. The horizontal lines represent the median of the data.

REFERENCES

1. Waggoner JJ, Pinsky BA. Zika virus: diagnostics for an emerging pandemic threat. J Clin Microbiol. 2016; 54(4): 860-7.

2. Oliveira JHM, Talyuli OAC, Goncalves RLS, Paiva-Silva GO, Sorgine MHF, Alvarenga PH, et al. Catalase protects *Aedes aegypti* from oxidative stress and increases midgut infection prevalence of Dengue but not Zika. PLoS Negl Trop Dis. 2017; 11(4): e0005525.

3. Fernandes RS, Campos SS, Ferreira-de-Brito A, Miranda RM, da Silva KAB, Castro MG, et al. *Culex quinquefasciatus* from Rio de Janeiro is not competent to transmit the local Zika virus. PLoS Negl Trop Dis. 2016; 10(9): e0004993.
